# A review of moxidectin vs. other macrocyclic lactones for prevention of heartworm disease in dogs with an appraisal of two commercial formulations

**DOI:** 10.3389/fvets.2024.1377718

**Published:** 2024-06-24

**Authors:** Kennedy Mwacalimba, Jenifer Sheehy, Christopher Adolph, Molly Savadelis, Kristina Kryda, Barbara Poulsen Nautrup

**Affiliations:** ^1^Outcomes Research, Zoetis, Parsippany, NJ, United States; ^2^Veterinary Professional Services, Zoetis, Parsippany, NJ, United States; ^3^Veterinary Medicine Research and Development, Zoetis, Kalamazoo, MI, United States; ^4^EAH-Consulting, Aachen, Germany

**Keywords:** moxidectin, macrocyclic lactone, heartworm, *Dirofilaria immitis*, prevention, resistance, compliance, dog

## Abstract

Macrocyclic lactones (MLs) are the only drug class currently licensed for heartworm disease prophylaxis. Macrocyclic lactones kill third- and fourth-stage larvae of *Dirofilaria immitis*, thus preventing the development of adult worms in dogs, which are responsible for heartworm disease, a potentially life-threatening condition. Despite considerable overlap in terms of endectocide spectrum, several important differences distinguish moxidectin from other MLs. Moxidectin has beneficial pharmacokinetic characteristics, such as a longer half-life and greater tissue distribution compared to ivermectin. Additionally, moxidectin has a greater margin of safety compared to ivermectin in dogs with ABCB1 (previously MDR1) gene-defect, which is commonly recognized in collies and other breeds. Multiple laboratory studies have shown that moxidectin is more effective than other commonly used heartworm preventives against resistant strains of *D. immitis*. This improved efficacy benefits individual dogs and helps reduce the risk of spreading resistant strains within the community. Despite the presence of proven resistant strains in the United States, non-compliance with preventive measures remains a major factor contributing to the diagnosis of heartworm disease in dogs. In retrospective analyses, the oral moxidectin combination product Simparica Trio^®^ (sarolaner, moxidectin, and pyrantel) was associated with increased compliance, resulting in more time of protection compared to dogs receiving flea/tick and heartworm preventive products separately. Compliance with the extended-release moxidectin injectables ProHeart^®^ 6 and ProHeart^®^ 12 was higher than with monthly heartworm preventives, as they provide 6 months or a full year of protection with one single injection, respectively, and revenues remain in the veterinary clinics as injectable moxidectin cannot be sourced through online retailers.

## 1 Introduction

**Figure 1:**
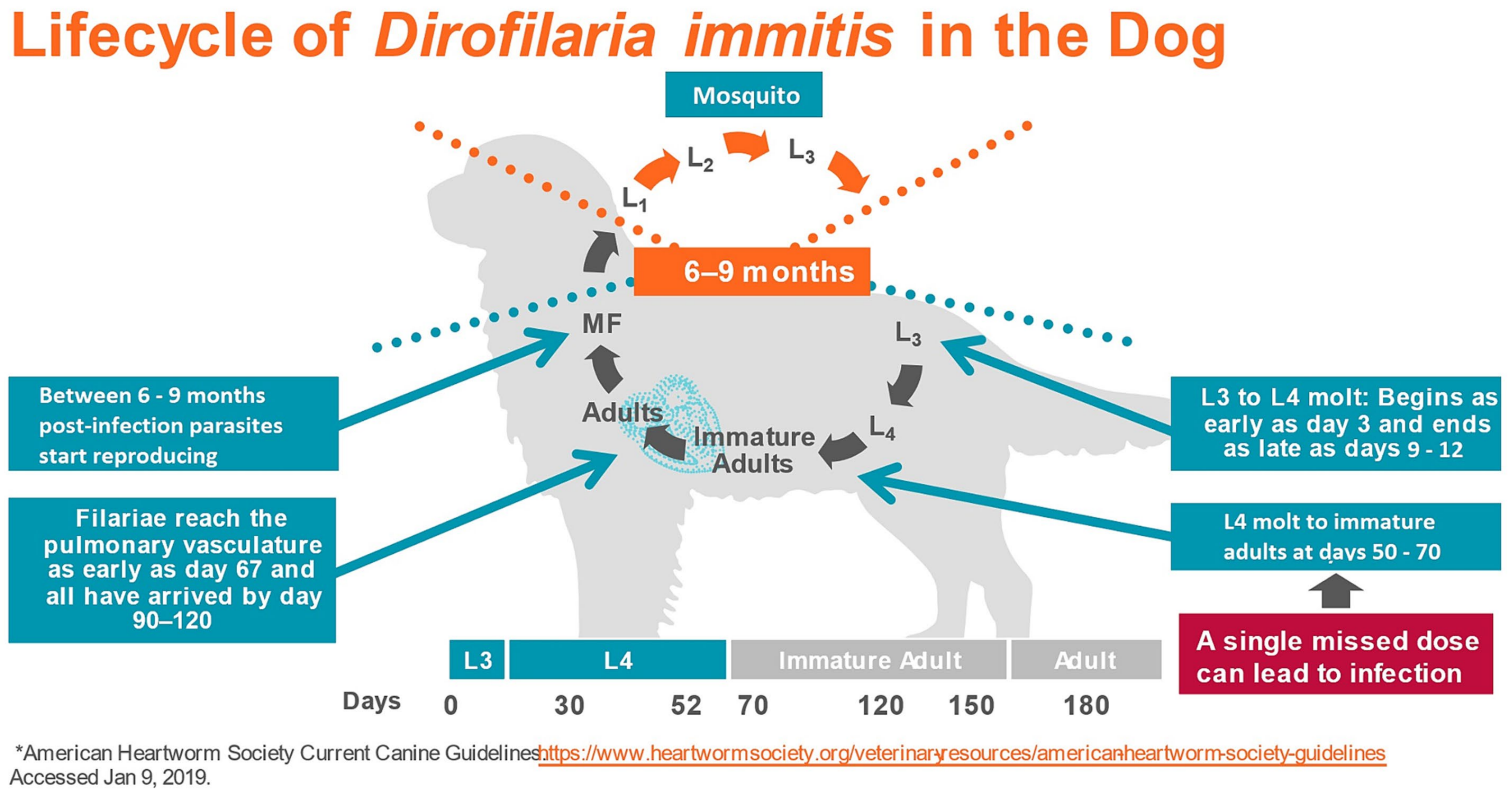
Lifecycle of *Dirofilaria immitis* in the dog (2) (L, Larvae; MF, Microfilariae).

*Dirofilaria immitis*, the causative agent of heartworm disease, is often considered the most important parasite of dogs in North America (1). The parasite is endemic in all US states (except Alaska) (2), in Canada (3), in many European countries (4, 5), Australia (6), and parts of Asia (7). *Dirofilaria immitis* is transmitted by the bite of infected mosquitoes. Over 70 species of mosquitoes have been identified as potential vectors of the filaroid parasite, although 10–12 of them may be the most important (8). During a blood meal from a heartworm-infected host, the mosquito ingests microfilariae, i.e., the stage that circulates in the bloodstream of infected animals after being produced by adult female heartworms. Microfilariae develop inside the arthropod to first-stage larvae (L1) and then molt twice to second (L2), and finally to the infective third-stage larvae (L3) in a period of 8–29 days, depending on the environmental temperature and mosquito species (9). The infective larvae migrate to the mouthparts of the mosquito and can be transmitted during another blood meal to a mammalian host, as they pass in a pool of mosquito hemolymph deposited at the site of the bite and enter the definitive host through the wound (9, 10). In the definitive host, L3 remain close to the site of inoculation and molt to the fourth-stage larvae (L4) within 3–5 days post-infection. The L4 then migrates in the subcutaneous and intramuscular tissues and finally molts to the juvenile adult stage between 50- and 58-day post-infection (11). By day 70, the first of the immature parasites arrive in the pulmonary artery (11) and by day 120, most have reached their final site of parasitism (12). Between 6 and 9 months after initial infection microfilariae appear in the bloodstream (11) (Figure 1).

Heartworm disease is characterized by the presence of adult worms in the pulmonary arteries. Although heartworm infection in dogs may remain subclinical, it often leads to clinical disease, mainly associated with pulmonary hypertension caused by structural changes in the arteries due to the presence of worms. With high worm burdens, worms can reside in the right atrium, right ventricle, and the vena cava, causing caval syndrome. Caval syndrome provokes valvular dysfunction, blood flow impairment, hemolysis, liver, kidney, and heart failure, and often results in the death of the dog (9, 13). The pathophysiologic impact of *D. immitis* mainly depends on the number of worms present in the pulmonary arteries and duration of infection, the size of the dog relative to number of adult heartworms, and the individual immune response to the infection (9).

Treating heartworm disease is expensive (14) and especially in moderate and severe infections or in patients with concurrent disease, often challenging (15). To maintain animal health and welfare, the mainstay of heartworm management is to prevent the development of adult nematodes, which are responsible for heartworm disease. This preventive approach relies on a single drug class, the macrocyclic lactones (ML), which kill the extremely sensitive L3/L4 stages in the mammalian host (1), and are the focus of this review. However, as the mosquito is an obligate intermediate host and vector for heartworms, the opportunity to interrupt the chain of transmission at the level of the vector should not be ignored by the pet owner and veterinarian (2, 16).

There are two subfamilies within the ML drug class, the avermectins and milbemycins. In veterinary medicine, ivermectin is the most used member of the avermectin subfamily, which also includes abamectin, doramectin, and selamectin. Moxidectin and milbemycin oxime are examples of commercially available milbemycins (17, 18). Commonly used MLs for heartworm prevention in dogs are oral formulations containing ivermectin, milbemycin oxime, or moxidectin (19). In several countries, including the United States and Australia, moxidectin is also available as an extended-release, long-acting injectable, which is indicated for annual (ProHeart^®^ 12 in the United States and ProHeart^®^ SR 12 in Australia, hereafter referred to as PH12) or biannual (ProHeart^®^ 6 in the United States, hereafter referred to as PH6) administration (20–22). Topical formulations of MLs for heartworm prophylaxis contain moxidectin or selamectin (23).

There is considerable overlap between moxidectin and the commercially available avermectins in terms of endectocide spectrum. However, important differences distinguish moxidectin and several reviews have been published, focusing on differences in pharmacodynamics, pharmacokinetics, resistance, and safety between moxidectin and other MLs (17, 19, 24, 25). Recently, new studies have become available investigating compliance and pharmacoeconomic aspects with the use of PH6, PH12, and the moxidectin combination product Simparica Trio^®^ (moxidectin, sarolaner, and pyrantel).

The objective of this review was to provide clinicians with a summary of the main differences between moxidectin and other MLs for the prevention of heartworm disease in dogs with an appraisal of Proheart^®^ and Simparica^®^ Trio. We focused on formulations available in the United States, although additional formulations of moxidectin and other MLs are available in Europe and other parts of the World. Those aspects, which have been described comprehensively in previous reviews, will be streamlined to provide a clearer overview and enhance understanding of the distinctions between moxidectin and other MLs. Newly published studies evaluating the compliance and pharmacoeconomic aspects with the injectable moxidectin and the oral moxidectin, sarolaner, and pyrantel product were supplemented. Given that noncompliance with heartworm preventive administration remains the primary contributing factor to the development of heartworm disease in dogs (26), it was essential to provide an updated comprehensive review summarizing all pertinent aspects of heartworm prevention for clinicians.

## 2 Pharmacology and toxicology

### 2.1 Pharmacodynamics

**Figure 2:**
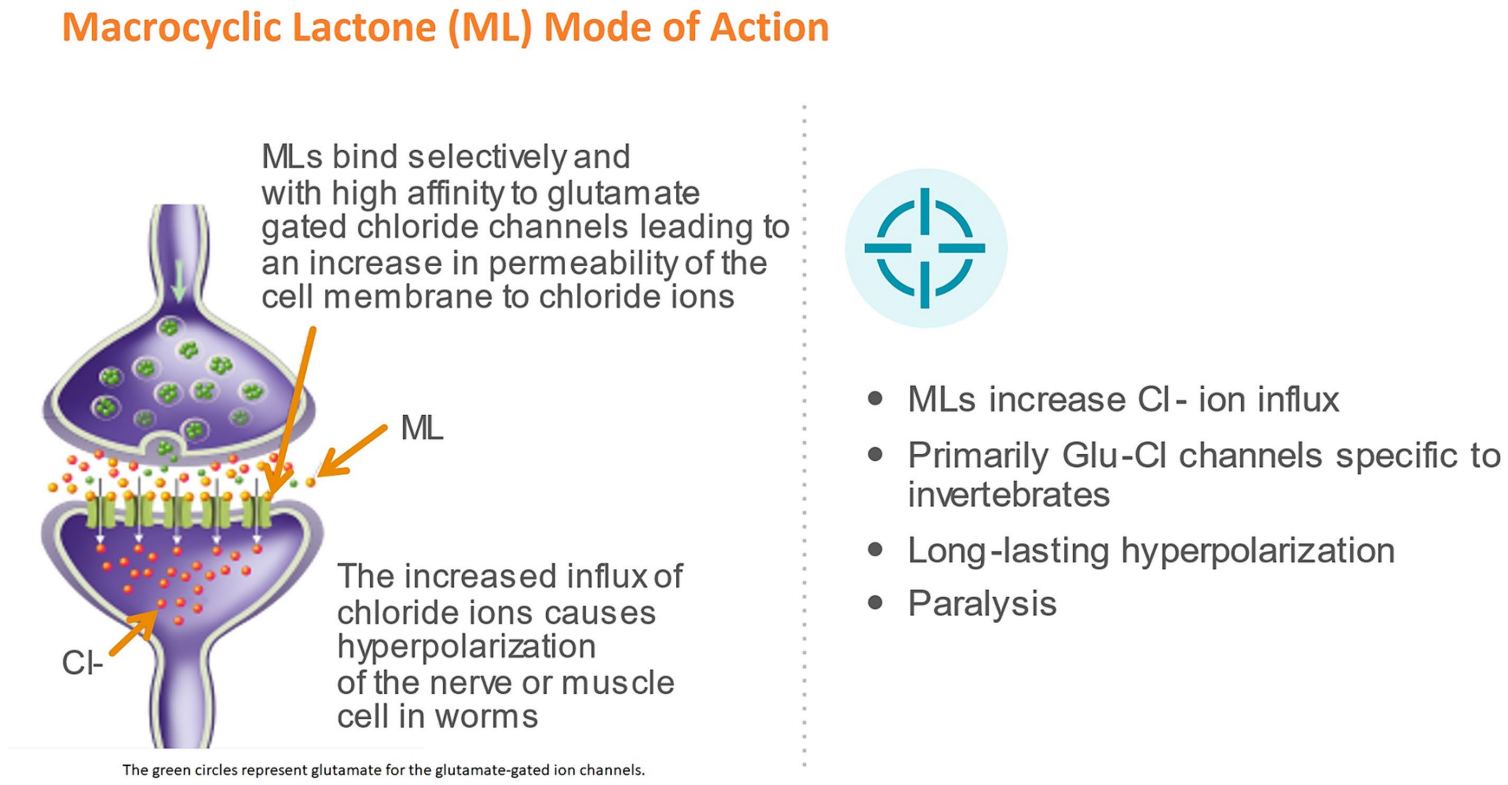
Mode of action of macrocyclic lactones (ML). The green circles represent glutamate for the glutamate-gated ion channels.

Avermectins and milbemycins have a common pharmacophore: a 16-member macrocyclic lactone ring fused with both benzofurane and spiroketal functions, which is recognized by specific chloride ion channel receptors (24). In nematodes, the MLs act by binding in a pseudo-irreversible manner to glutamate-gated chloride channels (GluCls), which is regarded as the main mechanism of action of this class of drugs (27–30). The GluCls are widely expressed in the nematode nervous system and pharyngeal muscles, but not in vertebrates, making them ideal drug targets for selective activity against parasites in mammals (24, 28). The binding of MLs opens the GluCl channel and increases the influx of Cl-ions, resulting in hyperpolarization and flaccid paralysis of neuromuscular systems in the nematode (31) (Figure 2). Studies have shown, however, that moxidectin and ivermectin do not interact with GluCls in the same way (32, 33). Structural differences, related to the presence and absence of various substitutes to the macrocyclic lactone ring, are at least partly responsible for this (24). It has been suggested that these differences may also have an impact on the efficacy of the drugs against resistant strains (33).

In contrast to the L3 and L4 stages, neither movement nor pharyngeal pumping are required for survival in adult filariae. In this stage MLs induce long-lasting reduction in the production of microfilariae (31). Data suggest that the main target of MLs, the GluCL, are also present in reproductive tissues of the adult filaroid parasite and it has been hypothesized that paralysis of muscles in these sites may reduce gamete production and embryogenesis in filarial worms, thereby explaining the observed suppression of microfilaria production following ML treatment (1, 34).

The extremely high potency of all macrocyclic lactones (MLs) against the L3 stage of *D. immitis in vivo* is not reflected in their efficacy *in vitro*. When L3 larvae are incubated *in vitro* with concentrations of ivermectin equivalent to those found in animals on ivermectin prophylaxis, there is little effect on the parasites’ motility or ability to migrate (35). A drug-stimulated attachment of canine peripheral immune cells to *D. immitis* has been reported, supporting the hypothesis that the host’s immune system plays an integral role in the killing of the parasite post-ML treatment *in vivo*, with MLs enhancing the immune response against the parasites (29, 35, 36), likely by inhibiting the secretion of immunomodulatory molecules that otherwise enables the parasite to escape from the host immune system (37).

Macrocyclic lactones also bind to gamma-aminobutyric acid (GABA) gated chloride channels. GABA receptors, however, seem to be a secondary target as they appear to be less sensitive than GluCl receptors in nematodes (17, 27). In mammals MLs bind to GABA_A_ receptors, which are widely expressed in the central nervous system (CNS) of vertebrates (38). The affinity to GABA_A_ channels varies among different MLs. Moxidectin has a lower affinity and potentiation *in vitro* compared to ivermectin, suggesting that moxidectin has a higher safety profile in mammals (39). In mammals, P-glycoproteins (P-gp) located at the blood–brain barrier normally prevent MLs from reaching GABA receptors in the CNS. P-glycoproteins are membrane transporters belonging to the ATP-binding cassette (ABC) superfamily, encoded by the ABCB1 (formerly known as MDR1) gene (40, 41). Toxicity of MLs in dogs has been associated with an accumulation of MLs in the CNS due to an overdose exceeding the transport capacity of P-gp or, in the case of a malfunctioning blood–brain barrier, due to genetic mutations (42) (see section Toxicology).

### 2.2 Pharmacokinetics

The concentration and length of time MLs are present in host tissues are important determinants of antiparasitic efficacy (17). Compared to other MLs, moxidectin is characterized by a much larger volume of distribution, a remarkably long mean residence time in host tissues, high plasma and lipid concentrations and a relatively large area under the plasma drug concentration–time curve (AUC) (24). In dogs, the terminal elimination half-life after oral administration of 250 μg/kg ivermectin and 250 μg/kg moxidectin was 3.3 days and 25.9 days, respectively (43) and peak plasma concentrations were 132.6 and 234.0 ng/mL for ivermectin and moxidectin, respectively. The area under the concentration-time curve in dogs was 5.6 μg h/mL (ivermectin) and 11.8 μg h/mL (moxidectin) and the mean residence time 98.4 and 696.6 h, respectively (43). Plasma drug concentration has been shown to be correlated to the ML disposition in tissues and is therefore considered a good predictor of anthelmintic efficacy for MLs (17).

Metabolism plays only a minor role in their elimination. MLs are primarily eliminated through efflux transporters as the parent substance in the host’s feces (17, 24, 44). In mice, ivermectin is actively excreted from the intestine via an active P-gp-dependent pathway, while moxidectin is mostly excreted via a P-gp-independent pathway at the intestinal barrier (45).

The higher lipophilicity of moxidectin compared to ivermectin contributes to its higher retention rate in fatty tissue and longer elimination half-life (17), but also permits convenient administration by different routes, including subcutaneous administration (24). The extended-release moxidectin injectables (PH6 and PH12) contain 10% moxidectin in glyceryl tristearate microspheres, which gradually erode after subcutaneous injection, liberating the active ingredient moxidectin over 6 or 12 months, respectively (46).

### 2.3 Toxicology

#### 2.3.1 Toxicity with macrocyclic lactones

Drugs of the avermectin and milbemycin classes have a wide margin of safety between therapeutic and toxic dosages when administered to companion animals at their labeled dosage and dosing frequency. Toxicity in dogs has been linked to ML accumulation in the CNS. This can occur due to an overdose that exceeds the transport capacity of P-gp or due to a malfunctioning blood–brain barrier caused by an ABCB1 gene defect (42). Accidental overdoses have been reported in dogs with no ABCB1 gene defect from miscalculation of a dosage when using a high concentrated large animal formulation off-label. Additionally, accidental exposure to remnants in a discarded tube of equine dewormer or to concentrated MLs present in the dung of large animals can potentially cause a clinically relevant overdose in dogs (42, 47). Macrocyclic lactone toxicosis has also been reported in three dogs with normal ABCB1 genotype after accidental oral administration of a topical endectocide (Advocate^®^) (48). The predominant symptoms in case of ML toxicosis are CNS signs such as ataxia, lethargy, coma, tremors, seizures, mydriasis, and blindness (42).

Macrocyclic lactone toxicity has been well-documented in dogs with an ABCB1 gene defect (ABCB1-1∆ dogs), although causal doses are above those labeled for heartworm prevention (42, 49, 50). The ABCB1 gene defect results in the production of a nonfunctional P-gp. With P-gp playing an important role in the blood–brain-barrier, MLs can accumulate in the brain of ABCB1-1∆ dogs, which would normally be removed by membrane transporters (40, 51). As a result, ABCB1-1∆ dogs become extremely susceptible to toxicosis at doses well below those tolerated by dogs with the wild-type ABCB1 gene. Dogs may be homozygous or heterozygous for the defect, with homozygous dogs being at greater risk of developing toxicosis from ML exposure (50). The presence of this mutation has been detected in several breeds, including Collie, Longhaired Whippet, Australian Shepherd, Border Collie, and the German Shepherd (52).

Several studies investigated the effects of different MLs in ABCB1-1∆ dogs. A study on collies, which had previously shown mild reactions to an ivermectin challenge (120 μg/kg body weight, 20 times the minimum effective dosage), revealed a similar sensitivity to milbemycin oxime when administered at a comparable overdose (10 mg/kg, also 20 times the minimum effective dosage). The authors concluded that both substances have similar margins of safety (53). Moxidectin administered orally up to 90 μg/kg, i.e., 30-fold the recommended heartworm prophylaxis dose of a previous oral formulation (no longer available in the United States) produced no signs of toxicosis in collies known to exhibit mild to severe reactions to 20 times the recommended dose of ivermectin (54). In similarly sensitive collies the topical administration of 5 times the maximum recommended dose of an imidacloprid/moxidectin topical solution did not produce any signs of toxicity (55). The injectable moxidectin formulation PH12 did not demonstrate any adverse reactions when administered up to 5 times the recommended dose to collies sensitive to a dose of 120 μg/kg ivermectin (46). The lower toxicity of moxidectin compared to ivermectin and other avermectins has been explained by moxidectin’s lower affinity to mammalian GABA_A_ receptors and mammalian P-gp, which makes moxidectin treatment less critically dependent on a fully functioning blood–brain barrier (17, 39).

#### 2.3.2 Safety of moxidectin extended-release injectable

An extended-release microsphere suspension formulation of moxidectin (PH6) was launched in June 2001 in the United States by the former manufacturer Fort Dodge Animal Health, having demonstrated the safety of PH6 as requested by the US Food and Drug Administration Center for Veterinary Medicine (FDA CVM) (56). Shortly after introduction, concerns were raised about severe anaphylactoid responses in the first 48 h post-treatment (46). Following a voluntary recall of PH6, the former manufacturer commissioned an epidemiological study to determine the incidence of potential adverse events associated with PH6 compared to two oral monthly heartworm preventives and/or vaccines (57). Medical records of a national network of 403 full-service primary care animal hospitals (Banfield, the Pet Hospital) in over 40 states were used. More than 6 million encounters for almost 2 million individual dogs were included in the analysis, covering the period between January 1, 2002, and August 31, 2004. The incidence of allergic reactions was similar for dogs that received PH6, any of the oral monthly heartworm preventives, or vaccine alone. However, the incidence of allergic reactions was consistently higher in vaccinated dogs compared with unvaccinated dogs, regardless of the heartworm preventive they received (57).

When PH12 was introduced into the United States market in 2019 it had already been registered in Australia since late 2000, where it has served as the leading heartworm preventive drug for nearly 2 decades (20, 46). For registration by the FDA CVM, PH12 had shown margin of safety in dogs being treated at 1, 1.5, and 2.5 times the recommended dosage three times at 6-month intervals (half of the licensed dosing interval), male and female reproductive safety, and safety in heartworm-positive dogs as well as in ivermectin-sensitive dogs (46, 58).

The safety of PH12 was also evaluated in a large multi-site clinical field study, involving 296 dogs treated with Heartgard^®^ Plus (ivermectin and pyrantel) and 297 dogs treated with PH12 over 20 months (59). Due to the long study duration of 20 months, most of the dogs experienced at least one abnormal clinical sign (85.1% ivermectin and pyrantel combination; 87.9% PH12). Most of the symptoms were consistent with sporadic occurrences of conditions commonly observed in the general dog population, with vomiting, lethargy, diarrhea, and anorexia being the adverse reactions reported most frequently (59). Hypersensitivity-related reactions considered likely related to treatment included one case for ivermectin and two cases for PH12, with all three being considered mild to moderate in severity. Both PH12 cases were successfully treated with antihistamine. At the second dosing a year later, one of these dogs was administered antihistamine prior to treatment. In both dogs, however, post-treatment response was uneventful (46). Because of the lack of a response after the second PH12 injection, it was assumed that the initial response was anaphylactoid rather than a Type 1 hypersensitivity. It is known that anaphylactoid reactions can be observed following the first administration of an agent and may not occur at subsequent exposures. Altogether it was concluded that the incidence of hypersensitivity-related responses for PH12 is in line with that of other marketed products, thereby being in accordance with experiences from non-United States markets where the product has been well accepted for decades (46).

## 3 Efficacy of macrocyclic lactones in view of resistant *Dirofilaria immitis* strains

### 3.1 Current situation of resistance

All licensed heartworm preventives in the United States had to demonstrate 100% efficacy at the time of approval (60, 61). However, as early as 1998, the FDA CVM received reports of suspected lack of efficacy of MLs, with the number of records increasing dramatically over the subsequent years (60). A retrospective medical record review revealed that the vast majority of the investigated cases of assumed lack of efficacy between 2004 and 2011 were due to non-compliance (26). However, in 1.7% of the cases, no purchase gaps or other factors indicating insufficient administration of the heartworm preventive were found (26). In 2011, it was reported for the first time that a single label dose of both ivermectin and milbemycin oxime failed to achieve 100% efficacy in dogs experimentally infected with an isolate (MP3) originally collected in 2007 from a naturally infected dog (62). In the same year, a controlled laboratory study reported efficacy rates <100% against *D. immitis* with three different heartworm preventives (ivermectin, milbemycin oxime, and selamectin) (63). The proof of unequivocal resistance was obtained in subsequent efficacy studies (64) and confirmed when evidence of genetic differences between less susceptible and susceptible isolates of *D. immitis* was found (65) and that this resistance is inherited between generations of *D. immitis* (66).

It is now believed that resistant strains of *D. immitis* might have been present prior to the use of MLs but were very rare (1). Until 2000 most heartworm preventive studies for MLs submitted to the FDA CVM were conducted using different generations of the same strain that had been isolated from a dog in the late 1960s (61). Some authors have raised concerns about whether the demonstrated 100% efficacy of MLs in licensing studies accurately represents the genetic and phenotypic diversity within the broader *D. immitis* population. This skepticism arises from the limited number of dogs included in the treatment and control groups of previous approval studies (1, 61).

To date, the existence of ML-resistant *D. immitis* populations in the United States is well documented (1, 9, 17, 19, 25). Resistance in *D. immitis* is still mainly identified in the Lower Mississippi River Valley (LMRV), a region where heartworm transmission is most intense, having resulted in strong recommendations to have all domesticated dogs on heartworm preventives year-round. The high availability of mosquito vectors, coupled with the enforced administration of MLs favors resistant parasite survival, while transmission of susceptible genotypes of *D. immitis* is truncated by constant treatment with heartworm preventives (1).

In Europe, to date, there have not been any confirmed resistant strains reported (9). Suspected cases have been investigated, but results have so far not confirmed resistance to MLs in Europe (67, 68). Although also suspected in Australia, genotypic assays have not yet unequivocally demonstrated the presence of ML-resistant *D. immitis* (6, 69).

The geographical distribution of heartworm disease appears to be expanding, in the United States as well as in Europe, which is due to changes in climate, increasing pet travel, and the expansion of mosquito vectors into new areas (4, 70, 71), thereby increasing the risk of spread of resistant strains of *D. immitis*.

### 3.2 Mechanism of resistance

The suggested mechanism of resistance has been previously reviewed (1, 17, 24, 72) and will be summarized here to help understand potential differences between moxidectin and other MLs. The efflux transporters P-gp have received the most attention when investigating the mechanism of resistance. P-glycoprotein is not only responsible for preventing MLs from entering the brain tissue of mammals, but also for controlling the tissue distribution of the drug in the whole organism (44, 72). Drug resistance in pathogens is frequently associated with alterations in drug transport via the upregulation of cellular efflux mechanisms, resulting in lower drug concentrations at the side of the relevant receptor(s) (72, 73). There is mounting evidence that repeated exposure to ivermectin can select for an overexpression of P-gp and other ABC transporters in nematodes, which decreases drug concentration at the target, thereby reducing the efficacy of ivermectin (72, 73). Moxidectin has been shown to interact differently with nematode P-gp and overexpression of P-gp was reduced with moxidectin as compared with ivermectin (17). The resistance to ivermectin was reversible by co-administration of P-gp-inhibitors, suggesting the involvement of P-gp in resistance against MLs (74, 75). Sensitivity to moxidectin was also increased in ivermectin-resistant strains following addition of P-gp inhibitors. However, this effect was not as strong as with ivermectin, likely due to moxidectin’s weaker binding affinity to P-gp (76).

It has been reported that parasites resistant to ivermectin show some degree but not complete cross-resistance to moxidectin (17). It was concluded that moxidectin selects less rapidly for resistance than avermectins and remains more potent than the avermectins against nematodes exhibiting resistance (17, 24). Although other mechanisms might be involved in resistant strains, such as the interaction with GluCl receptors (33), P-gp mechanisms are today assumed to play at least an early role in a step-wise process of resistance development (24).

With the proof of resistant strains of *D. immitis*, studies were conducted to find the genetic basis of resistance. Samples from parasites suspected to be ML-resistant, as well as those from non-resistant parasites, were analyzed for single-nucleotide polymorphisms (SNPs). Two SNPs in *D. immitis* genes encoding a P-gp transporter were found to be markedly elevated in phenotypically resistant strains. The SNPs reflected differences in nucleotide sequence at two positions, where G instead of A was found. In phenotypically resistant microfilariae the genotype GG-GG (homozygote in both positions) was significantly higher compared to non-resistant samples (77, 78). The observed homozygosity in these parasites was most likely due to ML selection as inbreeding was unlikely in worms being derived from different locations (77). It is, however, important to note that the observed GG-GG genotype most likely is only linked to the causative gene rather than being causative for the trait of ML resistance, as it was not present in all microfilariae that had survived ML treatment (78). Because of the high correlation of the GG-GG genotype with ivermectin resistance, it has been proposed to be a genetic marker (77). The identification of genetic markers is appropriate in research studies, but the technique is not appropriate for application in a clinic on individual cases of apparent breakthrough heartworm infection and new techniques and further research is warranted (1).

### 3.3 Efficacy studies

With the proof of resistance, multiple studies were conducted, investigating the efficacy of different MLs against various resistant strains.

Comparative efficacy studies involving moxidectin and other macrocyclic lactones (MLs) have been conducted in laboratory settings. In the following, this review focuses on direct comparisons of moxidectin and other MLs, as it has been shown that efficacy response between studies differ even when using the same strain of *D. immitis*. Differences in the efficacy response can be explained by normal variability across studies and the genetic bottlenecking that occurs with subsequent passaging of generations of the same parasite strains (61, 79). Therefore, we abstained from indirectly comparing outcomes of different MLs versus placebo using the same resistant strain, but only included direct comparisons of the moxidectin formulations with other MLs.

#### 3.3.1 Laboratory efficacy studies

Seven studies evaluated the comparative efficacy of different heartworm preventive medications under laboratory conditions (63, 64, 79–81), including at least one formulation of moxidectin. These studies also included a negative control group to calculate preventive efficacy. In most studies, Heartgard® Plus (ivermectin/pyrantel) and Interceptor® Plus (milbemycin oxime/praziquantel) served as positive controls. These were compared with either the extended-release moxidectin injectable PH12 (79), the oral moxidectin combination product Simparica Trio® (moxidectin, sarolaner, and pyrantel) (80), or moxidectin dosed at 24 μg/kg (the minimum dose in Simparica Trio®) (81). Two studies specifically investigated the efficacy of Advantage® Multi (topical moxidectin) compared to ivermectin, milbemycin oxime, and selamectin in various combination formulations (63, 64). All drugs were administered according to approved label recommendations and repeated at 30-day intervals, except for PH12, which provides protection for 12 months. The resistant strains of *D. immitis* used in these studies were ZoeLA (80, 81), JYD-34 (64, 79, 81), or MP3 (63). In one study, 100 L3 larvae were inoculated (63), while in all other studies, 50 L3 larvae were used. More information is included in Table 1.

**Table 1:** Efficacy of moxidectin and other commonly used macrocyclic lactones against resistant strains of *Dirofilaria immitis* in laboratory studies: effects on geometric mean worm counts.

Reference	Strain of *D. immitis*	Day of inoculation^†^	Day of necropsy	Active ingredient against *D. immitis*	Dosage	Product	No. of dogs	Route of admin.	No. of treatments	No. of dogs with worms	Geometric mean worm counts	Preventive efficacy (%)
McTier et al. (79)—Study 1	JYD-34	−30	185	**Moxidectin**	**0.5 mg/kg**	**ProHeart® 12**	**5**	**s.c.**	**1**	**0**	**0.0** ^**a**^	**100**
				Ivermectin	≥ 6 μg/kg	Heartgard® Plus	6	oral	6	6	26.8^b^	10.5
				Milbemycin oxime	≥ 0.5 mg/kg	Interceptor® Plus	6	oral	6	6	25.5^b^	14.6
				Negative control	n.a.	n.a.	6	n.a.	0	6	29.9^b^	n.a.
McTier et al. (79)—Study 2	JYD-34	165	360	**Moxidectin**	**0.5 mg/kg**	**ProHeart® 12**	**6**	**s.c.**	**1**	**4**	**0.6** ^**a**^	**98.3**
				Ivermectin	≥ 6 μg/kg	Heartgard® Plus	6	oral	12	6	21.7^b^	37.7
				Milbemycin oxime	≥ 0.5 mg/kg	Interceptor® Plus	6	oral	12	6	22.7^b^	34.9
				Negative control	n.a.	n.a.	**6**	**n.a.**	**0**	**6**	34.9^c^	n.a.
Myers et al. (80)	ZoeLA	−30	241	**Moxidectin**	**≥ 24 μg/kg**	**Simparica Trio®**	**6**	**Oral**	**6**	**5**	**1.0** ^**a**^	**97.2**
				Ivermectin	≥ 6 μg/kg	Heartgard® Plus	6	Oral	6	6	32.5^b^	8.5
				Milbemycin oxime	≥ 0.5 mg/kg	Interceptor® Plus	6	Oral	6	6	22.8^b^	35.9
				Negative control	n.a.	n.a.	6	n.a.	0	6	35.5^b^	n.a.
Kryda et al. (81)—Study 1	ZoeLA	−30	243	**Moxidectin**	**24 μg/kg**	**Same dosage as in Simparica® Trio**	**6**	**Oral**	**4**	**5**	**1.1** ^**a**^	**96.8**
				**Moxidectin**	**24 μg/kg**		**6**	**Oral**	**6**	**5**	**1.4** ^**a**^	**96.1**
				Ivermectin	≥ 6 μg/kg	Heartgard® Plus	6	Oral	6	6	29.0^b^	18.7
				Milbemycin oxime	≥ 0.5 mg/kg	Interceptor® Plus	6	Oral	6	6	28.1^b^	21.2
				Negative control	n.a.	n.a.	6	n.a.	0	6	35.6^b^	n.a.
Kryda et al. (81)—Study 2	JYD-34	−30	237	**Moxidectin**	**24 μg/kg**	**Same dosage as in Simparica® Trio**	**6**	**Oral**	**4**	**5**	**1.3** ^**a**^	**95.9**
				**Moxidectin**	**24 μg/kg**		**6**	**Oral**	**6**	**2**	**0.2** ^**b**^	**99.3**
				Ivermectin	≥ 6 μg/kg	Heartgard® Plus	6	Oral	6	6	11.9^c^	63.9
				Milbemycin oxime	≥ 0.5 mg/kg	Interceptor® Plus	6	Oral	6	6	14.9^c^	54.6
				Negative control	n.a.	n.a.	6	n.a.	0	6	32.9^d^	n.a.
Blagburn et al. (64)	JYD-34	−30	124–126	**Moxidectin**	**≥ 2.8 mg/kg**	**Advantage Multi®**	**8**	**Topical**	**1**	**0**	0.0*	**100**
				Ivermectin	> 6 μg/kg	Heartgard® Plus	8	Oral	3	8	13.1	29.0
				Milbemycin oxime	≥ 0.5 mg/kg	Trifexis®	8	Oral	3	8	8.8*	52.2
				Selamectin	> 6 mg/ kg	Revolution®	8	Topical	3	8	13.1*	28.8
				Negative control	n.a.	n.a.	8	n.a.	0	8	18.4	n.a.
Blagburn et al. (63)	MP3	−30	119–120	**Moxidectin**	**> 3 mg/kg**	**Advantage Multi®**	**8**	**Topical**	**1**	**0**	0.0*	**100**
				Ivermectin	> 6 μg/kg	Heartgard® Plus	8	Oral	1	7	2.3*	95.6
				Milbemycin oxime	> 0.5 mg/kg	Interceptor®	8	Oral	1	7	2.4*	95.4
				Selamectin	> 6 mg/kg	Revolution®	8	Topical	1	7	2.3*	95.5
				Negative control	n.a.	n.a.	8	n.a.	0	8	51.6	n.a.

^†^First treatment = day 0. ^a,b,c^Geometric mean counts with different superscript letters within a study are statistically significant (*p* < 0.05). ^*^Statistically significant difference (*p* < 0.05) vs. negative control.

In the two studies investigating PH12, the preventive efficacy of the extended-release moxidectin injectable was 100 and 98.3% against the resistant strain JYD-34, while the efficacy of ivermectin and milbemycin oxime was between 10.5 and 37.7%. The geometric mean worm counts in dogs receiving PH12 were significantly lower than those in dogs receiving Heartgard^®^ Plus, Interceptor^®^ Plus or no heartworm prevention (79).

Three studies investigated the preventive efficacy of oral moxidectin using either the combination product Simparica Trio^®^ (80) or granules which were of the same composition as the moxidectin component of Simparica Trio^®^ (81). Over all studies, preventive efficacy of moxidectin against resistant strains was between 95.9 and 99.3%, while the efficacy of ivermectin and milbemycin was between 8.5 and 63.9%. Dogs receiving oral moxidectin had significantly lower geometric mean worm counts than dogs receiving Heartgard^®^ Plus, Interceptor^®^ Plus or no heartworm prevention (80, 81). It should be noted that the oral moxidectin dose in all studies was ≥24 μg/kg, which was considered the optimal dose in a dose-finding study on resistant *D. immitis* strains (82). A dose of 12 μg/kg moxidectin administered once, for example, resulted in less than two third of the efficacy of one dose of 24 μg/kg moxidectin against the resistant strain JYD-34, while three consecutive doses of 24 μg/kg provided ≥ 98.8% efficacy for JYD-34, ZoeLA, and ZoeMO resistant strains (82). These results, however, cannot be extrapolated to a new combination product (licensed for a moxidectin dose of 12 μg/kg) that has been proven effective at preventing dirofilariosis when administered for at least 6 months (83).

In the two studies investigating topical moxidectin, preventive efficacy in dogs receiving Advantage^®^ Multi was 100%, while the efficacy of ivermectin, milbemycin oxime, and selamectin was 29, 52.5, and 28.8% (*D. immitis* strain JYD-34) (64) and 95.6, 95.4, and 95.5% (*D. immitis* strain MP3), respectively (63). All results are displayed in Table 1.

Five of the seven studies also reported the number of dogs testing positive for heartworm antigen and microfilariae at different time points (79–81). At the final evaluation time point, one dog receiving the extended-release moxidectin injectable PH12 tested positive for heartworm antigen in one of two studies, whereas all (6 of 6) or most (5 of 6) dogs receiving ivermectin, milbemycin oxime, or no heartworm preventive tested positive for heartworm antigen and microfilariae at the final evaluation time points in the two studies.

When considering dogs receiving the same number of moxidectin treatments as dogs receiving Heartgard^®^ Plus or Interceptor^®^ Plus (i.e., six consecutive doses), the number of dogs testing positive for heartworm antigen or microfilariae at any time point was between 0 and 2 (out of six dogs per study and time point), whereas the corresponding numbers in dogs receiving ivermectin, milbemycin oxime or no heartworm preventive were between 4 and 6 out of six dogs per study and time point (Table 2).

**Table 2:** Efficacy of moxidectin and other commonly used macrocyclic lactones against resistant strains of *Dirofilaria immitis* in laboratory studies: effects on antigen test and micofilariae.

Reference	Strain of *D. immitis*	Day of inoculation^†^	Active ingredient against *D. immitis*	Dosage	Product	Route of admin.	No. of treatments	Time point (study day)	No. of dogs positive for HW AG test	No. of dogs positive for MF
McTier et al. (79)—Study 1	JYD-34	−30	**Moxidectin**	**0.5 mg/kg**	**ProHeart® 12**	**s.c.**	**1**	**151**	**0 of 5**	**0 of 5**
			Ivermectin	≥ 6 μg/kg	Heartgard® Plus	Oral	6	151	5 of 6	5 of 6
			Milbemycin oxime	≥ 0.5 mg/kg	Interceptor® Plus	Oral	6	151	6 of 6	6 of 6
			Negative Control	n.a.	n.a.	n.a.	0	151	6 of 6	4 of 6
			**Moxidectin**	**0.5 mg/kg**	**ProHeart® 12**	**s.c.**	**1**	**178**	**0 of 5**	**0 of 5**
			Ivermectin	≥ 6 μg/kg	Heartgard® Plus	Oral	6	178	6 of 6	6 of 6
			Milbemycin oxime	≥ 0.5 mg/kg	Interceptor® Plus	Oral	6	178	6 of 6	6 of 6
			Negative Control	n.a.	n.a.	n.a.	0	178	6 of 6	6 of 6
McTier et al. (79)—Study 2	JYD-34	165	**Moxidectin**	**0.5 mg/kg**	**ProHeart® 12**	**s.c.**	**1**	**331**	**0 of 6**	**0 of 6**
			Ivermectin	≥ 6 μg/kg	Heartgard® Plus	Oral	12	331	6 of 6	0 of 6
			Milbemycin oxime	≥ 0.5 mg/kg	Interceptor® Plus	Oral	12	331	6 of 6	0 of 6
			Negative Control	n.a.	n.a.	n.a.	0	331	6 of 6	0 of 6
			**Moxidectin**	**0.5 mg/kg**	**ProHeart® 12**	**s.c.**	**1**	**360**	**1 of 6**	**0 of 6**
			Ivermectin	≥ 6 μg/kg	Heartgard® Plus	Oral	12	360	6 of 6	5 of 6
			Milbemycin oxime	≥ 0.5 mg/kg	Interceptor® Plus	Oral	12	360	6 of 6	5 of 6
			Negative Control	n.a.	n.a.	n.a.	0	360	6 of 6	5 of 6
Myers et al. (80)	ZoeLA	−30	**Moxidectin**	**≥ 24 μg/kg**	**Simparica Trio®**	**Oral**	**6**	**180**	**1 of 6**	**0 of 6**
			Ivermectin	≥ 6 μg/kg	Heartgard® Plus	Oral	6	180	6 of 6	6 of 6
			Milbemycin oxime	≥ 0.5 mg/kg	Interceptor® Plus	Oral	6	180	6 of 6	6 of 6
			Negative Control	n.a.	n.a.	n.a.	0	180	6 of 6	6 of 6
			**Moxidectin**	**≥ 24 μg/kg**	**Simparica Trio®**	**Oral**	**6**	**210**	**2 of 6**	**0 of 6**
			Ivermectin	≥ 6 μg/kg	Heartgard® Plus	Oral	6	210	6 of 6	6 of 6
			Milbemycin oxime	≥ 0.5 mg/kg	Interceptor® Plus	Oral	6	210	6 of 6	5 of 6
			Negative Control	n.a.	n.a.	n.a.	0	210	6 of 6	6 of 6
			**Moxidectin**	**≥ 24 μg/kg**	**Simparica Trio®**	**Oral**	**6**	**236**	**2 of 6**	**0 of 6**
			Ivermectin	≥ 6 μg/kg	Heartgard® Plus	Oral	6	236	6 of 6	5 of 6
			Milbemycin oxime	≥ 0.5 mg/kg	Interceptor® Plus	Oral	6	236	6 of 6	5 of 6
			Negative Control	n.a.	n.a.	n.a.	0	236	6 of 6	6 of 6
Kryda et al. (81)—Study 1	ZoeLA	−30	**Moxidectin**	**24 μg/kg**	**Same dosage as in Simparica® Trio**	**Oral**	**4**	**210**	**3 of 6**	**2 of 6**
			**Moxidectin**	**24 μg/kg**		**Oral**	**6**	**210**	**0 of 6**	**2 of 6**
			Ivermectin	≥ 6 μg/kg	Heartgard® Plus	Oral	6	210	6 of 6	5 of 6
			Milbemycin oxime	≥ 0.5 mg/kg	Interceptor® Plus	Oral	6	210	6 of 6	6 of 6
			Negative Control	n.a.	n.a.	n.a.	0	210	6 of 6	6 of 6
			**Moxidectin**	**24 μg/kg**	**Same dosage as in Simparica® Trio**	**Oral**	**4**	**243**	**4 of 6**	**1 of 6**
			**Moxidectin**	**24 μg/kg**		**Oral**	**6**	**243**	**0 of 6**	**0 of 6**
			Ivermectin	≥ 6 μg/kg	Heartgard® Plus	Oral	6	243	6 of 6	5 of 6
			Milbemycin oxime	≥ 0.5 mg/kg	Interceptor® Plus	Oral	6	243	6 of 6	5 of 6
			Negative Control	n.a.	n.a.	n.a.	0	243	6 of 6	6 of 6
Kryda et al. (81)—Study 2 (84)	JYD-34	−30	**Moxidectin**	**24 μg/kg**	**Same dosage as in Simparica® Trio**	**Oral**	**4**	**180**	**0 of 6**	**0 of 6**
			**Moxidectin**	**24 μg/kg**		**Oral**	**6**	**180**	**0 of 6**	**0 of 6**
			Ivermectin	≥ 6 μg/kg	Heartgard® Plus	Oral	6	180	4 of 6	4 of 6
			Milbemycin oxime	≥ 0.5 mg/kg	Interceptor® Plus	Oral	6	180	4 of 6	6 of 6
			Negative Control	n.a.	n.a.	n.a.	0	180	6 of 6	6 of 6
			**Moxidectin**	**24 μg/kg**	**Same dosage as in Simparica® Trio**	**Oral**	**4**	**210**	**3 of 6**	**1 of 6**
			**Moxidectin**	**24 μg/kg**		**Oral**	**6**	**210**	**1 of 6**	**0 of 6**
			Ivermectin	≥ 6 μg/kg	Heartgard® Plus	Oral	6	210	5 of 6	5 of 6
			Milbemycin oxime	≥ 0.5 mg/kg	Interceptor® Plus	Oral	6	210	5 of 6	6 of 6
			Negative Control	n.a.	n.a.	n.a.	0	210	6 of 6	6 of 6
			**Moxidectin**	**24 μg/kg**	**Same dosage as in Simparica® Trio**	**Oral**	**4**	**236**	**5 of 6**	**1 of 6**
			**Moxidectin**	**24 μg/kg**		**Oral**	**6**	**236**	**1 of 6**	**0 of 6**
			Ivermectin	≥ 6 μg/kg	Heartgard® Plus	Oral	6	236	6 of 6	5 of 6
			Milbemycin oxime	≥ 0.5 mg/kg	Interceptor® Plus	Oral	6	236	6 of 6	6 of 6
			Negative Control	n.a.	n.a.	n.a.	0	236	6 of 6	6 of 6

s.c., subcutaneous; HW AG, Heartworm antigen; MF, Microfilariae. ^†^First treatment = day 0.

#### 3.3.2 Field studies

Two multi-center field trials (59, 84) evaluated the comparative efficacy of moxidectin and ivermectin (Table 3). In both studies, compliance was rigorously documented. In the first study, 593 dogs from 19 veterinary clinics across the United States were enrolled (59). All animals completing the 605-day study received either 20 consecutive doses of Heartgard^®^ Plus (ivermectin + pyrantel) or two doses of PH12, resulting in a total of 218 and 235 evaluable cases for the monthly heartworm preventive and the extended-release moxidectin formulation, respectively. Efficacy parameters were number of dogs positive for heartworm antigen and number of dogs positive for microfilariae at days 365, 480, and 605. No dog treated with PH12 tested positive for heartworm antigen or microfilariae on any of the study test days. In the ivermectin group, four dogs tested positive for heartworm antigen (three dogs on day 365 and one dog on day 480) with three of these dogs having also tested positive for microfilariae (two dogs on day 218 and one dog on day 480) (Table 3). All microfilariae positive dogs were from the LMRV. A total of 101 evaluable cases were from LMRV, and these dogs were distributed evenly through both treatment groups (52 dogs in the PH12 group and 49 dogs in the ivermectin group) (59).

**Table 3:** Effect of moxidectin compared to other commonly used macrocyclic lactones on *Dirofilaria immitis* antigen test and micofilariae in field studies.

Reference	Active ingredient against *D. immitis*	Product	Route of administration	Study day^†^	No. of dogs positive for HW AG test	No. of dogs positive for MF
McTier et al. (59)	**Moxidectin**	**ProHeart® 12**	**s.c.**	**365**	**0 of 235**	**0 of 235**
	Ivermectin	Heartgard® Plus	Oral	365	3 of 218	2 of 218
	**Moxidectin**	**ProHeart® 12**	**s.c.**	**480**	**0 of 226**	**0 of 226**
	Ivermectin	Heartgard® Plus	Oral	480	1 of 209	1 of 209
	**Moxidectin**	**ProHeart® 12**	**s.c.**	**605**	**0 of 222**	**0 of 222**
	Ivermectin	Heartgard® Plus	Oral	605	0 of 201	0 of 201
Kryda et al. (84)	**Moxidectin**	**Simparica Trio®**	**Oral**	**330**	**0 of 246**	**0 of 246**
	Ivermectin	Heartgard® Plus	Oral	330	2 of 119	1 of 219

s.c., subcutaneous; HW AG, Heartworm antigen; MF, Microfilariae. ^†^First treatment = day 0. ProHeart 12 was administered at days 0 (59, 84) and 365 (84). Oral heartworm preventives were administered monthly over 20 (59) or 11 (84) months.

In the second study, 410 client-owned dogs were enrolled from 23 veterinary clinics across the United States (84). The dogs received 11 consecutive doses of either oral moxidectin (24 μg/kg) administered in combination with sarolaner and pyrantel or the ivermectin combination product Heartgard^®^ Plus (ivermectin + pyrantel). In total, 365 dogs (246 dogs in the moxidectin and 119 dogs in the ivermectin group, with a similar percentage geographic distribution between the two groups) were included in the efficacy evaluation. No dogs in the moxidectin-treated group tested positive for heartworm antigen or microfilariae, whereas two dogs in the ivermectin group tested heartworm antigen positive, with one of these dogs also testing microfilariae positive (Table 3). All dogs tested positive were from the LMRV (84). The development of patent infections in dogs from two separate studies under natural *D. immitis* exposure while receiving Heartgard^®^ Plus with confirmed compliance, strongly suggests that these dogs were infected with ML-resistant heartworm strains.

It should be noted that sarolaner, one of the three compounds in the moxidectin combination product Simparica Trio, has demonstrated high efficacy against *Aedes aegypti*, one of the main mosquito species responsible for the worldwide transmission of mosquito-borne pathogens, including *D. immitis*. The high insecticidal efficacy of sarolaner has the potential to reduce the mosquito population prior to a new feeding on the same or new host (16), thereby contributing to a multimodal approach to prevent both heartworm disease development and heartworm transmission to improve outcomes for both individual dogs and the population at large (2).

## 4 Compliance

While it is documented that resistant heartworm strains exist, owner compliance rather than product failure is still the major cause of heartworm disease diagnosed in dogs (2, 26, 85). It is estimated that only one third of all medicalized dogs in the United States receive one or more doses of heartworm preventives annually (70). It is possible for an animal to become infected because of only one skipped or delayed preventive dose, particularly in highly endemic areas (2, 26). Nevertheless, achieving good owner compliance remains an ongoing challenge for veterinarians (86). Cost and administration have been reported as important barriers for the success of heartworm prophylaxis (87). The method of administration is a general factor affecting compliance in pets, as owners have to be confident and capable of administering a spot-on or administering a tablet. If owners have concerns over their ability to administer preventives, there is a greater risk of non-compliance (88).

Studies investigating the compliance of moxidectin formulations compared with other heartworm preventives using purchase transaction data, have been conducted by Zoetis, the manufacturer of PH6, PH12, and the moxidectin combination product Simparica Trio^®^ (20–22, 89). The use of transaction data has limitations as outlined in a previous study (89). However, owner self-reported data on compliance is fraught with bias and can be unreliable, whereas transactional data are an objective measurement (90). Transaction data confirmed the low compliance rate with heartworm preventives in the dog population in the United States, as an average of only 25.7% of all canine patients seen at the included clinics received some sort of heartworm protection during the 2-year examination period (20).

A recent study evaluated the purchase compliance with the combination product Simparica Trio^®^ (moxidectin + sarolaner + pyrantel) compared to flea/tick and heartworm products being purchased separately. Dogs with transactions for Simparica Trio^®^ received an average 7.2 months of protection against flea/tick and heartworm over the 12 months observation period, whereas dogs with transactions for flea/tick and heartworm products separately were on average protected for only 4.4 and 6.8 months, respectively (89). In the United States, another flea/tick and heartworm combination products has become available (Nexgard^®^ PLUS, containing afoxolaner, moxidectin, and pyrantel pamoate) (91), and a combination product of afoxolaner and milbemycin oxime (Nexgard Spectra^®^) is marketed in European countries (92) and Australia (93). However, we are not aware of a study evaluating the compliance with one of these combination products.

Further studies evaluated the compliance of the extended-release moxidectin injectables (PH6 and/or PH12) compared to monthly heartworm preventives in the United States (22, 89) and Australia (21). All studies grouped heartworm preventives that needed to be given monthly together, i.e., did not differentiate between oral or topical products. During the observation period 2014–2017, the 6-month injectable PH6 resulted in a higher annual compliance than monthly heartworm preventives, as the proportion of dogs that received two injections was 51.7% compared to 24.4% of dogs with two transactions for six doses of monthly heartworm preventives (22). Another study compared the monthly heartworm equivalent doses of the two injectable moxidectin formulations (PH6 and PH12) with the number of monthly heartworm preventive doses dispensed annually. Between September 2019 and August 2020, dogs that received monthly heartworm preventives had on average purchase records for 7.3 months, whereas dogs that received PH6 were on average 8.2 monthly protected. ProHeart 12 offered a full year of protection (12 monthly equivalent doses) with a single injection (89). In the Australian study, the mean single-year purchase compliance over the 5-year observation period was 96.3% for dogs treated with PH12 and 28.7% for dogs dispensed monthly heartworm preventives. In Australia, PH12 is registered for use in dogs from 12 weeks of age. Continuous protection in these young dogs, however, requires two injections of PH12 by the time a dog is 15 months of age. Since not all young dogs received the second injection within the 15 months period, the mean annual compliance was slightly below 100% (21). In the United States, PH12 is licensed in dogs older than 12 months, followed by annually repeated administrations (58), thus corresponding to an annual compliance of 100% in all dogs from first injection.

## 5 Pharmacoeconomic analyses

Four studies conducted by the manufacturer of PH6, PH12, and the moxidectin combination product Simparica Trio® evaluated the economic benefits of various ML formulations to veterinarians by analyzing transaction data (20–22, 89). The same study that compared the purchase compliance of Simparica Trio^®^ to flea/tick and heartworm preventives being dispensed separately, also evaluated the economic outcome for the veterinarian. The lower time of annual protection with flea/tick and heartworm products dispensed separately was mirrored in lower annual revenues generated on average with these drugs per dog ($145.52) compared to average annual revenues with Simparica Trio^®^ ($189.17 per dog), which provided more time of protection. Dogs receiving the combination therapy also generated more revenues (+ $57.45) through additional services per dog compared to dogs on dual therapy (89).

Three studies evaluated the revenue benefits when using the extended-release moxidectin injectables (PH6 and PH12) compared to other heartworm preventives (20–22). In an Australian study, it was shown that dogs on PH12 or monthly heartworm preventives generate more revenue not only through heartworm purchases but also for other products and services compared to dogs not dispensed heartworm preventives. Despite buying fewer months of protection with monthly heartworm preventives annual costs per pet owner for monthly heartworm preventives were higher than costs for PH12 which provided a full year of protection. However, monthly heartworm preventives often provided protection against additional parasites, which was reflected in the relative costs (21). A United Sates study evaluated revenues generated in dogs receiving PH6 compared with dogs dispensed monthly heartworm preventives or monthly combination products (22). ProHeart 6 costs were similar to the dose equivalent of monthly heartworm preventives (i.e., 6 months) and cost less than six doses of monthly heartworm products with additional protection against parasites. A higher proportion of dogs on PH6 received additional products and services compared to dogs on monthly heartworm preventives and the proportion of heartworm preventives on total revenues were 29.7, 31.0, and 55.6% for PH6, monthly heartworm preventives, and monthly heartworm preventive combinations, respectively (22). Another United States study reported a higher increase in heartworm preventive revenues in new PH12 users, outpacing the overall clinic revenue increases between the first and second annual period. In contrast, increases in heartworm preventive revenues in clinics not having implemented PH6 or PH12 were below the percentage increase of overall clinic revenues, although the costs of annual doses of monthly heartworm preventives were similar to costs of PH12 (20).

At the time of these studies, moxidectin was the sole macrocyclic lactone (ML) available as an extended-release injectable formulation. The studies concluded that the use of extended-release injectables, specifically PH6 and PH12, ensures that pharmacy revenues for heartworm prevention remain within veterinary practices, as injectable moxidectin is not available through online retailers. Additionally, clinic visits are always associated with a veterinary examination, allowing the veterinarian to identify potential health issues and to ask about other preventive care that might be needed (20, 21), thereby not only contributing to the net clinic profit but also potentially adding benefits to the health and wellbeing of the dogs.

## 6 Conclusion

Resistance of some strains of *D. immitis* to MLs has been confirmed in the United States, but can also occur at any time in other countries. Moxidectin has demonstrated both a wider margin of safety and enhanced efficacy against tested resistant strains of *D. immitis*. Consequently, its utilization not only benefits individual dogs but also aids in mitigating the risk of resistant strain dissemination to the broader canine population. Despite the occurrence of resistant strains, lack of compliance is still the major cause of heartworm disease diagnosed in dogs. In retrospective analyses, the oral moxidectin combination product Simparica Trio^®^ was associated with better compliance, resulting in more time of protection compared to dogs receiving flea/tick and heartworm products separately. Adherence to extended-release moxidectin injectables PH6 and PH12 exhibited superior performance compared to purchase compliance with monthly heartworm preventives. These formulations offer extended protection durations of 6 months or a full year with a single injection, thereby contributing to enhanced revenue retention within veterinary clinics, as injectable moxidectin is not accessible through online retailers.

## Author contributions

KM: Writing – review & editing, Validation, Conceptualization. JS: Writing – review & editing, Validation. CA: Writing – review & editing, Validation. MS: Writing – review & editing, Validation. KK: Writing – review & editing, Validation. BPN: Writing – original draft.
